# Isolation of cancer‐associated fibroblasts and its promotion to the progression of intrahepatic cholangiocarcinoma

**DOI:** 10.1002/cam4.1704

**Published:** 2018-07-31

**Authors:** Meng Sha, Seogsong Jeong, Bi‐jun Qiu, Ying Tong, Lei Xia, Ning Xu, Jian‐jun Zhang, Qiang Xia

**Affiliations:** ^1^ Department of Liver Surgery Ren Ji Hospital School of Medicine Shanghai Jiao Tong University Shanghai China

**Keywords:** α‐SMA, cancer‐associated fibroblasts, intrahepatic cholangiocarcinoma, isolation, lymph node metastasis, lymphangiogenesis

## Abstract

Intrahepatic cholangiocarcinoma is a highly fatal tumor characterized by an abundant stromal environment. Cancer‐associated fibroblasts play key roles in tumor growth and invasiveness and have been regarded as a potential therapeutic target. This study was designed to isolate human primary cancer‐associated fibroblasts of intrahepatic cholangiocarcinoma to study tumor‐stroma interactions and to analyze the clinical relevance of alpha‐smooth muscle actin ‐positive cancer‐associated fibroblasts in patients with intrahepatic cholangiocarcinoma. The isolated cancer‐associated fibroblasts were positive for alpha‐smooth actin, fibroblast‐specific protein‐1, fibroblast activation protein, and PDGFR‐β. In addition, cancer‐associated fibroblasts were found to increase proliferation, migration, and invasion of cholangiocarcinoma cells in vitro and promote tumor growth of mice in vivo. Moreover, alpha‐smooth muscle actin‐positive expression of cancer‐associated fibroblasts predicted unfavorable prognosis in patients with intrahepatic cholangiocarcinoma and showed correlation with presence of lymph node metastasis. This study may provide a useful tool to investigate further effect of cancer‐associated fibroblasts on the molecular mechanism of cholangiocarcinoma cells as well as contribution of cancer‐associated fibroblasts in lymphangiogenesis and lymph node metastasis.

## 1 INTRODUCTION

Intrahepatic cholangiocarcinoma (ICC) is the second most common primary hepatic malignancy that accounts for approximately 10%‐15% of all primary liver cancers.^1^, ^2^ It is a highly aggressive cancer with poor prognosis and limited therapeutic strategies. Hepatic resection remains the potential curative treatment that provides patients to prolong survival outcomes.^3^ However, 5‐year survival rates of patients who received hepatic resection are barely at 30%‐35%.^4^, ^5^ For locally advanced or metastatic cholangiocarcinoma, although chemotherapy protocol with cisplatin plus gemcitabine was found to provide a significant survival advantage compared with gemcitabine alone, the median overall survival was no longer than 1 year.^6^ Moreover, the application of other therapies, such as radiotherapy and liver transplantation, is yet to be conclusively confirmed whether these therapies could provide better survival outcomes.^7^

Abundant stroma is the representative histological hallmark of ICC, and cancer‐associated fibroblasts (CAFs) are the most prominent stromal components, which play pivotal roles in modulating tumor microenvironment.^8^, ^9^ On the one hand, cancer cells were able to recruit CAFs and stimulate fibroblast migration in cholangiocarcinoma cells, which reconstructs stromal microenvironment and favors tumor progression.^10^ On the other hand, CAFs interact with other cells, including endothelial cells and inflammatory cells,^11^ and secrete a variety of soluble factors, such as cytokines and growth factors.^12^, ^13^ Furthermore, CAFs also activate numerous intracellular signaling pathways to promote tumor growth and invasiveness.

Cancer‐associated fibroblasts are morphologically identified by the typical spindle shape expressing α‐smooth muscle actin (α‐SMA) and other markers. The presence of α‐SMA‐positive CAFs has been reported to be correlated with shorter overall and tumor‐free survival rate in various types of carcinomas.^14^, ^15^, ^16^ Previously, there have been a few studies describing isolation of stromal cells derived from noncholangiocarcinoma samples.^17^, ^18^ However, there are limited studies about isolation of CAFs from human ICC tissues; therefore, the crosstalk between tumor cells and stroma still remains to be comprehensively identified.

This study was conducted to isolate and culture CAFs from surgically resected human ICC samples and to preliminarily investigate the interactions between CAFs and neoplastic cholangiocytes. In addition, we evaluated the clinical significance of α‐SMA‐positive CAFs in patients with intrahepatic cholangiocarcinoma.

## 2 MATERIALS AND METHODS

### 2.1 Study patients

From January 2007 to July 2015, consecutive patients with pathological confirmation of ICC who underwent liver resection at Renji Hospital (Shanghai, China) were enrolled in this study. Only the patients with single type of tumor without preoperative neoadjuvant therapies were included. Finally, a total of 106 patients were met the inclusion criteria. All patients underwent radical hepatic resection (surgical margin ≥ 2 cm). This study was approved by the local ethics committee of Renji Hospital, School of Medicine, Shanghai Jiao Tong University and conducted according to the Ethical Guidelines for Human Genome/Gene Research enacted by the Chinese Government and the Helsinki Declaration.

### 2.2 Isolation and culture of CAFs and normal fibroblasts

Cancer‐associated fibroblasts were isolated from 12 tumor samples of patients who demonstrated abundant stroma around neoplastic cholangiocytes. Normal liver fibroblasts (NFs) were isolated from specimens of nontumorigenic tissue areas. Both cancer and nontumorigenic specimens were washed three times with PBS, then minced into approximately 1‐2 mm^2^ sized pieces and digested using 10 mL of a 0.1% trypsin solution (Gibco, CA, USA) with 0.75 μg/μL collagenase I (Sigma Chemical Co., St. Louis, MO, USA). After variable digestion time, the homogenate was collected and passed through a 70 mmol/L strainer. Then, CAFs and NFs were purified using magnetic activated cell sorting (MACS). Cells were incubated with anti‐fibroblast microbeads (Miltenyi Biotec, Auburn, CA, USA) for 30 minutes. After passing through a MiniMACS separator, positive cells were resuspended and plated in RPMI 1640, 1% penicillin/streptomycin, 10% FBS (Gibco). After 24 hours culture on the first day, the medium was replaced to eliminate floating cells, thereafter, purified CAFs and NFs of ICC were obtained.

### 2.3 Immunofluorescence staining

Cancer‐associated fibroblasts of intrahepatic cholangiocarcinoma were fixed with 4% paraformaldehyde and incubated with α‐SMA (rabbit monoclonal; 1:100; Abcam, Cambridge, UK), FSP‐1 (rabbit monoclonal; 1:100; Abcam, Cambridge, UK), FAP (rabbit monoclonal; 1:100; Abcam, Cambridge, UK), and PDGFR‐β (rabbit monoclonal; 1:100; Abcam, Cambridge, UK) overnight at 4°C. CAFs were then incubated with associated Alex Fluor 594‐conjugated anti‐rabbit IgG for 1 hour. Nuclei were counterstained with DAPI.

### 2.4 Immunohistochemistry and evaluation

Four‐micrometer‐thick formalin‐fixed, paraffin‐embedded sections were cut and deparaffinized through xylene and ethanol. The sections were incubated overnight at 4°C with the primary antibodies against α‐SMA (rabbit monoclonal; 1:200; Abcam), FSP‐1 (rabbit monoclonal; 1:200; Abcam), FAP (rabbit monoclonal; 1:250; Abcam), and PDGFR‐β (rabbit monoclonal; 1:200; Abcam). The sections were then incubated with the appropriate biotinylated secondary antibodies and the immune complexes were then visualized using 3,3′‐diaminobenzidine. Stromal expression of α‐SMA in spindle‐shaped cells was defined as positive when over than 10% of CAFs were stained. All sections were evaluated independently by two investigators without any knowledge of the clinical features.

### 2.5 Preparation of conditioned medium

Conditioned medium derived from CAFs and NFs was used for proliferation, migration, and invasion in cancer cell lines. After isolated CAFs and NFs were plated and allowed to attach to cell culture dishes for 24 hours, the cells were rinsed twice with PBS and then incubated in RPMI 1640 with no FBS for another 48 hours. The conditioned medium derived from CAFs and NFs was harvested, centrifuged at 200 *g* for 10 minutes to remove cell debris, and added with 10% FBS. The CAFs and NFs‐conditioned medium (CAFs‐CM and NFs‐CM) were stored at −20°C until use.

### 2.6 Proliferation, migration, and invasion assays

Cell proliferation was measured using a Cell Counting Kit‐8 (CCK‐8). Viability of cells was measured using a Cell Counting Kit‐8. Briefly, 10 μL of CCK‐8 solution was added to each well with cholangiocarcinoma cells cultured in CAFs‐CM, NFs‐CM and medium with 10% FBS after 1, 2, 3, 4 and 5 days for proliferation measurement, respectively. In viable cells, 450 nm OD value was detected using a Spectra Max M2 spectrophotometer.

Migration and invasion of cultured cancer cells were assessed by counting the number of cells migrating or invading through uncoated or matrigel‐coated transwell chambers. Cancer cells (1 × 10^4^ cells) were seeded in the upper chambers, incubated with CAFs‐CM in the lower chambers as experiment groups, compared with NFs‐CM as controls. Cancer cells at the lower surface of the membrane were fixed with 70% ethanol, stained with crystal violet, and counted in five random fields. Each experiment was carried out in triplicate wells and independent experiments were repeated for three times.

### 2.7 Mouse xenografting experiments

Male SCID mice, 4 weeks of age were purchased from the Chinese Academy of Sciences (Shanghai, China). All animal experiments were approved by the Animal Care and Use Committee of Shanghai Jiaotong University School of Medicine. HuCCT‐1 cells (1 × 10^6^ cells) suspended in 0.2 mL of phosphate buffer (PBS) were implanted subcutaneously with or without 1 × 10^6^ CAFs into the flank of the mice. Tumor growth was followed with a caliper every 7 days since inoculation, and tumor volume (*V*) was calculated as follows: *V* = *ab* ^2^π/6, where *a* is the longest and *b* is the shortest of two perpendicular diameters. After 5 weeks, animals were sacrificed. Tumor and liver were removed, fixed in 10% formalin and paraffin‐embedded or immediately frozen in liquid nitrogen.

### 2.8 Real‐time PCR

Total RNA was extracted with the RNeasy Mini Kit (Qiagen, Hilden, Germany) according to the manufacturer's instructions and quantified by spectrophotometry (Nanodrop 2000; Thermo Scientific, Wilmington, DE, USA). Subsequently, total RNA was reverse‐transcribed with the RevertAid First Strand cDNA Synthesis Kit (Thermo Fisher Scientific). The real‐time PCR was performed using a CFX96 Touch Real‐Time PCR Detection System (Bio‐Rad Laboratories, Hercules, CA, USA). Expression of β‐actin served as internal control for normalization of signals.

### 2.9 Statistical analysis

The correlation between stromal expression for α‐SMA in CAFs and the clinicopathologic factors were evaluated using the chi‐square test. Survival curves were estimated using the Kaplan‐Meier method, and difference was analyzed using the log‐rank test. To evaluate independent prognostic factors associated with survival, the Cox regression hazard model was used to perform univariate and multivariate analysis. All statistical analysis was performed using software (SPSS, version 19.0, Chicago, IL, USA). Statistical significance was defined as *P *<* *0.05.

## 3 RESULTS

### 3.1 Primary CAFs and NFs from ICC

**Figure 1:**
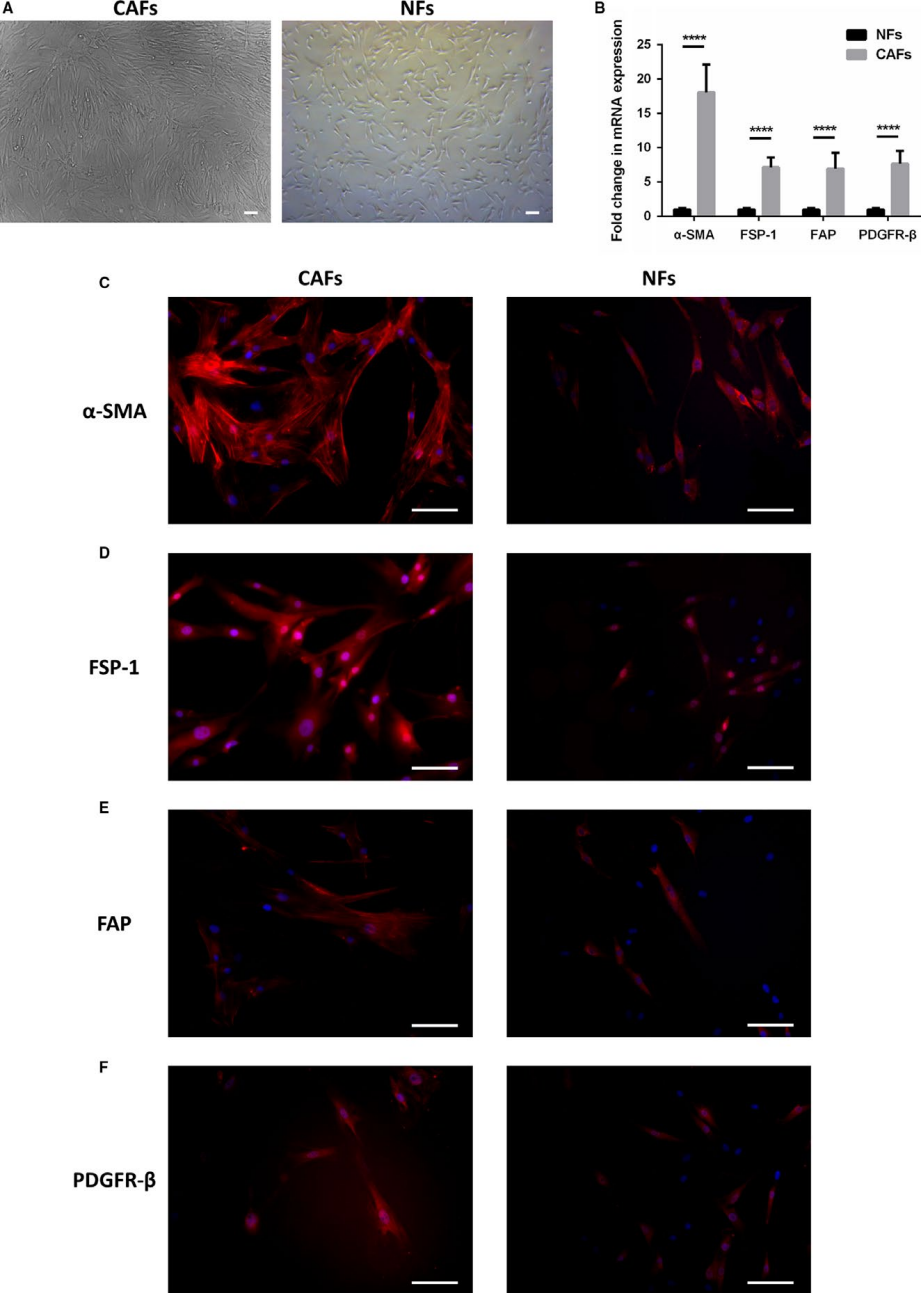
Generation of CAFs of human ICC. A, Isolated CAFs had irregular spindle‐shaped or stellate‐like morphology and formed the structure of network progressively, while isolated NFs were identified by the regular short spindle shape (scale bar: 100 μm). B, Relative mRNA level of α‐SMA, FSP‐1, FAP, and PDGFR‐β, measured by real‐time PCR in isolated CAFs and NFs (*P *<* *0.0001 versus expression in NFs). Bars represent the mean ± SD of triplicate experiments. Representative images of immunofluorescence staining of cultured CAFs and NFs with α‐SMA (C), FSP‐1 (D), FAP (E), and PDGFR‐β (F) antibodies (red) and counterstained with DAPI (blue; scale bar: 100 μm)

In order to better study the interaction between CAFs and cancer cells, we isolated CAFs from fresh ICC specimens through the digestion of trypsin and collagenase I. Primary CAFs and NFs mixed with other cell types and debris were purified through the method of magnetic activated cell sorting, which modifies the traditional technique of selective digestion and growth.^19^, ^20^ The CAFs and NFs shown in the experiments and figures were from a 52‐year‐old male patient who died 6 months after hepatic resection because of tumor recurrence. The clinicopathological characteristics of the patient were described in Table 1. As illustrated in Figure 1A, isolated CAFs had irregular spindle‐shaped or stellate‐like morphology and formed the structure of network progressively, while isolated NFs were identified by the regular short spindle shape. Real‐time PCR analysis for fibroblast markers demonstrated that the relative mRNA level of α‐SMA, FSP‐1, FAP, and PDGFR‐β in isolated CAFs was significantly elevated compared with NFs (Figure 1B). In addition, immunofluorescence staining of cultured CAFs and NFs showed that CAFs were strong positive for α‐SMA, FSP‐1, FAP, and PDGFR‐β with typical morphology, while these markers were low expressed in part of NFs (Figure 1C‐F).

**Table 1:** Clinicopathological characteristics of the selected patient with ICC to isolate CAFs

Clinicopathological characteristics	Value
Age (years)	52
Gender	Male
HBV infection	Present
Preoperative AFP (ng/mL)	<9
Preoperative CA19‐9 (U/mL)	>35
Tumor size (mm)	80
Tumor number	Single
Vascular invasion	Present
Lymph node metastasis	Present
Histological grade	Poor
Overall survival time (months)	6
Recurrence‐free survival time (months)	2

### 3.2 Immunostaining characteristics of CAFs in human tissue

**Figure 2:**
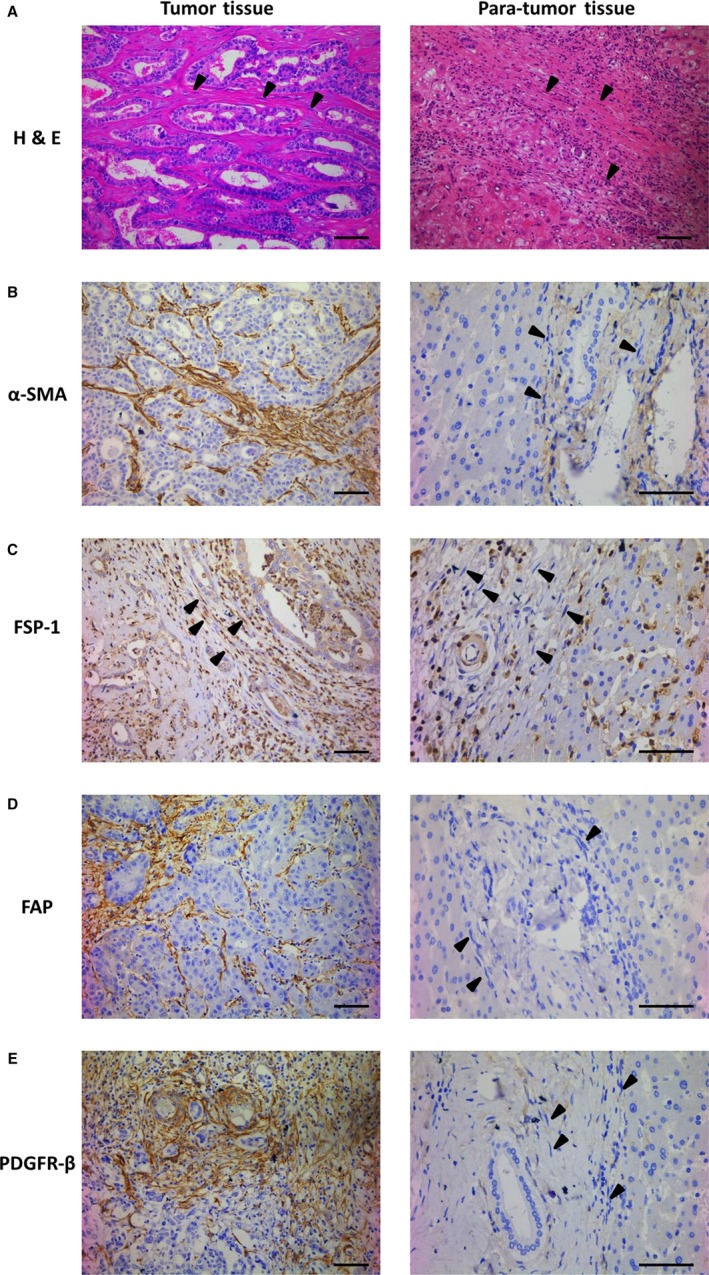
Expression of α‐SMA, FSP‐1, FAP, and PDGFR‐β in human tumor tissue of ICC and para‐tumor tissue. A‐E, Representative immunohistochemical images of human tumor tissue and para‐tumor tissue stained with H&E (A), α‐SMA (B), FSP‐1 (C), FAP (D), and PDGFR‐β (E) antibodies. CAFs around cancer cells were positive stained for α‐SMA, FSP‐1, FAP, and PDGFR‐β in tumor tissue of ICC, while NFs were negative stained in nontumorigenic tissue (arrowheads; scale bar: 100 μm)

To further identify the characteristics of CAFs in ICC, immunohistochemical staining for fibroblast markers was performed in both human tumor tissue and para‐tumor tissue (Figure 2). Stromal expression of α‐SMA, FSP‐1, FAP, and PDGFR‐β were positive stained in CAFs around cancer cells. In contrast, NFs were negative for α‐SMA, FSP‐1, FAP, and PDGFR‐β in para‐ nontumorigenic tissue. These results reveal the characteristics of CAFs, a type of activated fibroblasts positive expressing specific markers compared with NFs.

### 3.3 CAFs promote proliferation, migration, and invasion of cholangiocarcinoma cells in vitro

**Figure 3:**
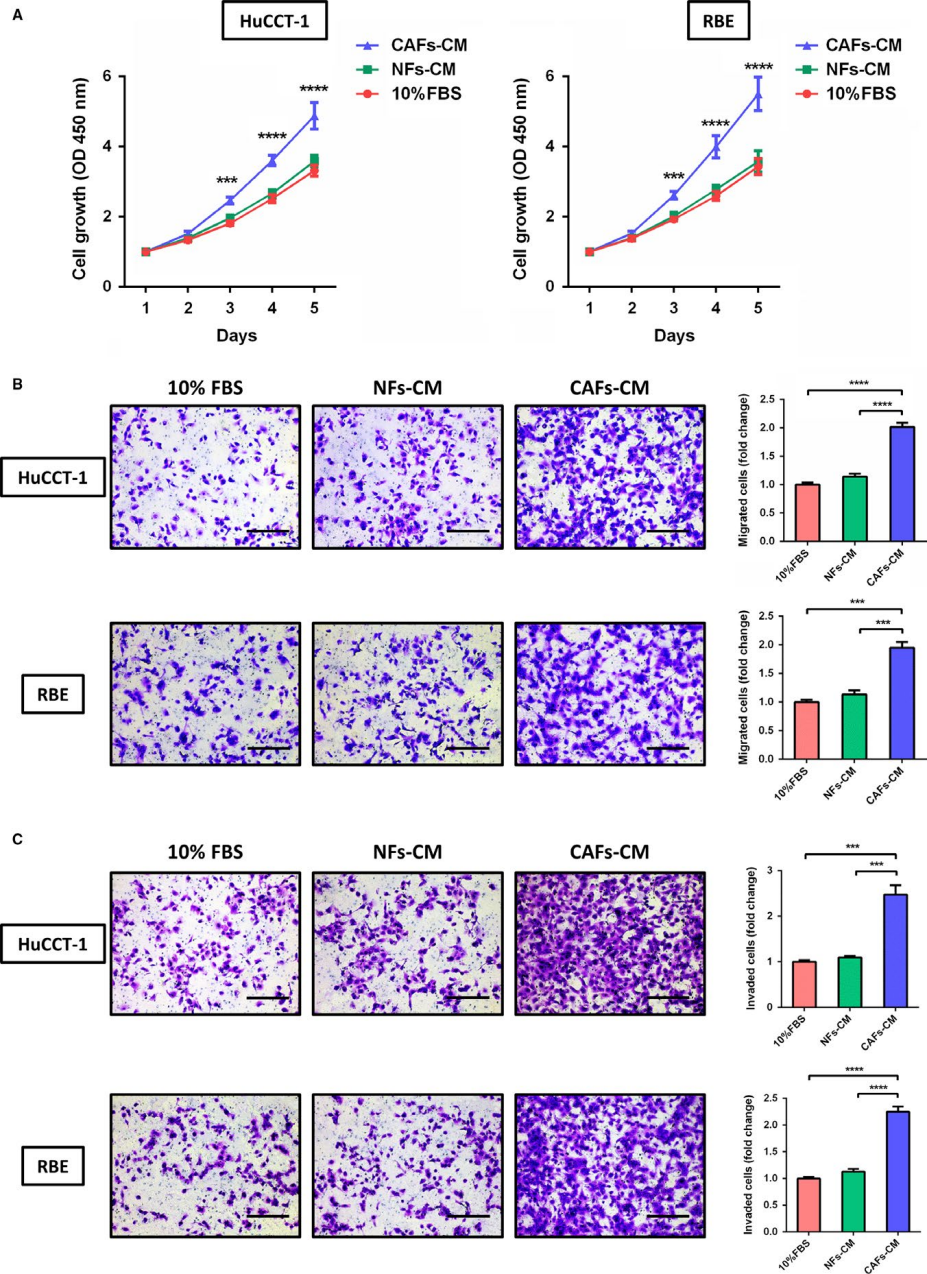
CAFs promote proliferation, migration, and invasion of cholangiocarcinoma cells in vitro. A, The growth of cholangiocarcinoma cells (HuCCT‐1 and RBE) cultured with CAFs‐conditioned medium (CAFs‐CM) was greatly accelerated compared with cells cultured with normal fibroblasts‐conditioned medium (NFs‐CM) and 10% FBS medium, especially on day 3 (*P *<* *0.001), day 4 (*P *<* *0.0001), and day 5 (*P *<* *0.0001). B, CAFs significantly promoted the migration of cholangiocarcinoma cells (*P *<* *0.0001 for HuCCT‐1 and *P *<* *0.001 for RBE; scale bar: 200 μm). C, The invasion ability of cholangiocarcinoma cells was severely promoted when cultured with CAFs‐CM compared to NFs‐CM and 10% FBS medium (*P *<* *0.0001 for both HuCCT‐1 and *P *<* *0.001 for RBE; scale bar: 200 μm). Bars represent the mean ± SD of triplicate experiments

To determine the interaction between CAFs and cholangiocarcinoma cells, growth‐promoting effect of CAFs on cholangiocarcinoma cells with different CMs was examined by CCK8 assay. As showed in Figure 3A, cancer cells of ICC cultured in CAFs‐conditioned medium (CAFs‐CM) grew faster as compared with cells cultured in NFs‐conditioned medium (NFs‐CM) and 10% FBS medium. The difference was significant on day 3 (*P *<* *0.001), day 4 (*P *<* *0.0001) and day 5 (*P *<* *0.0001) for both cell lines of HuCCT‐1 and RBE. It suggested that CAFs had the potential of accelerating the growth of cholangiocarcinoma cells. Migration and invasion assay of cholangiocarcinoma cells were further performed. As the representative staining results shown in Figure 3B, it was demonstrated that compared to control cells treated with NFs‐CM and 10% FBS medium, the migration ability of cholangiocarcinoma cells cultured in CAFs‐CM were significantly promoted (*P *<* *0.0001 for HuCCT‐1 and *P *<* *0.001 for RBE). In addition, a significant increase was also observed in invading cholangiocarcinoma cells cultured with CAFs‐CM compared with NFs‐CM and 10% FBS medium (Figure 3C; *P *<* *0.001 for HuCCT‐1 and *P *<* *0.0001 for RBE). The results revealed the promoting role of CAFs in cholangiocarcinoma cells migration and invasion.

### 3.4 CAFs promote cholangiocarcinoma tumor growth in vivo

**Figure 4:**
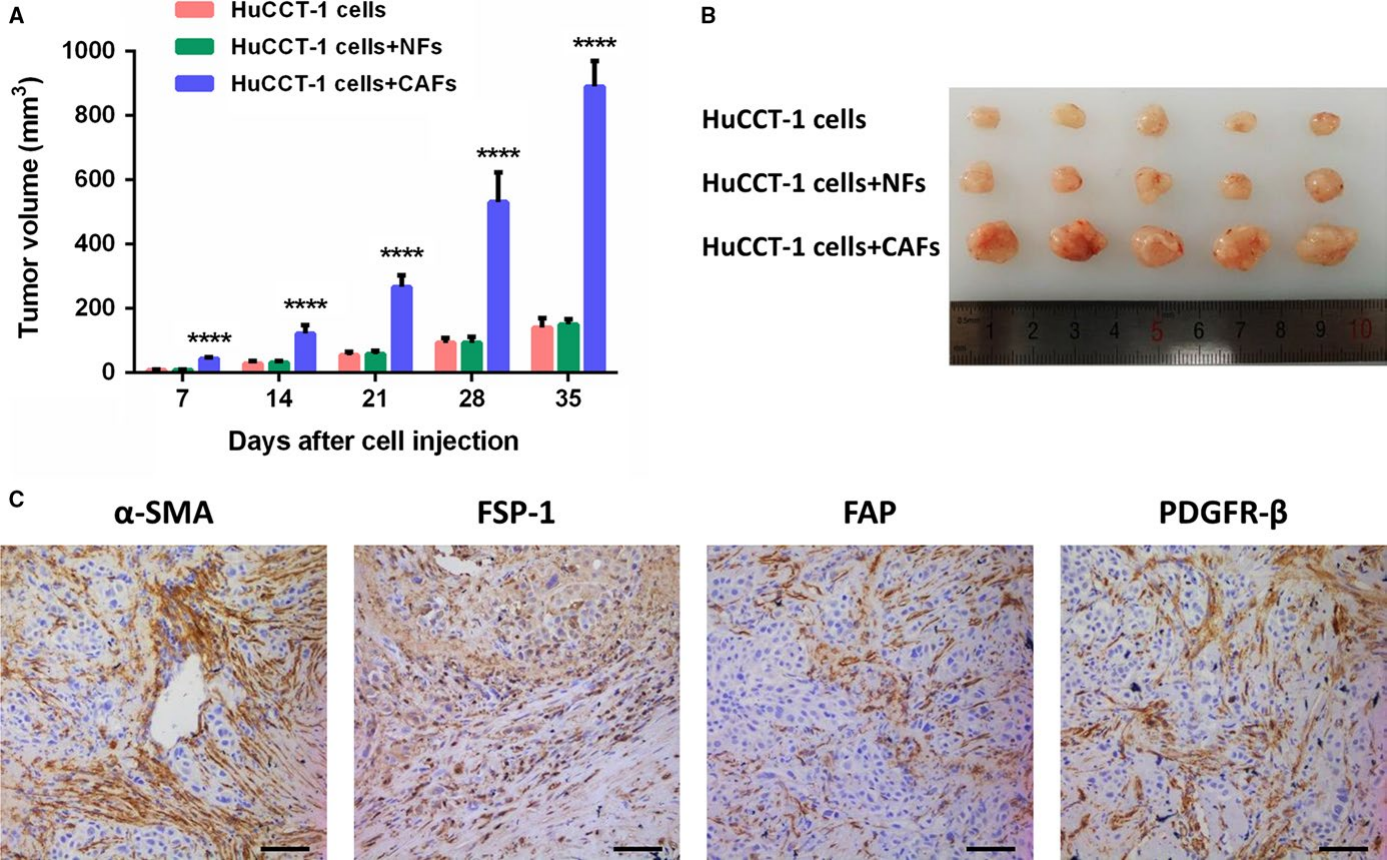
CAFs promote cholangiocarcinoma tumor growth in vivo. A, Tumor volume of mice bearing cholangiocarcinoma cells (HuCCT‐1), cholangiocarcinoma cells plus NFs, and cholangiocarcinoma cells plus CAFs (*P *<* *0.0001 versus tumor of cholangiocarcinoma cells and cholangiocarcinoma cells plus NFs). Bars represent the mean ± SD. B, Representative image of tumor from each group at sacrifice. C, Representative immunohistochemical stainings for α‐SMA, FSP‐1, FAP, and PDGFR‐β of mice tumor sections (scale bar: 100 μm)

To determine the contribution of CAFs on cholangiocarcinoma biology, we performed coinjection experiments of cholangiocarcinoma cells (HuCCT‐1) with primary CAFs isolated from ICC in a subcutaneous xenograft model. Cholangiocarcinoma cells were injected subcutaneously with or without CAFs into mice. CAFs promoted tumor growth every week postcell injection with an average 5‐fold increase in tumor volume compared with mice injected with cholangiocarcinoma cells plus NFs or cholangiocarcinoma cells alone (Figure 4A,B). It is also demonstrated that tumors developed in xenograft mice were similar to human ICC, showing stromal expression of α‐SMA, FSP‐1, FAP, and PDGFR‐β.

### 3.5 Correlation of α‐SMA expression in CAFs with clinicopathological parameters

**Figure 5:**
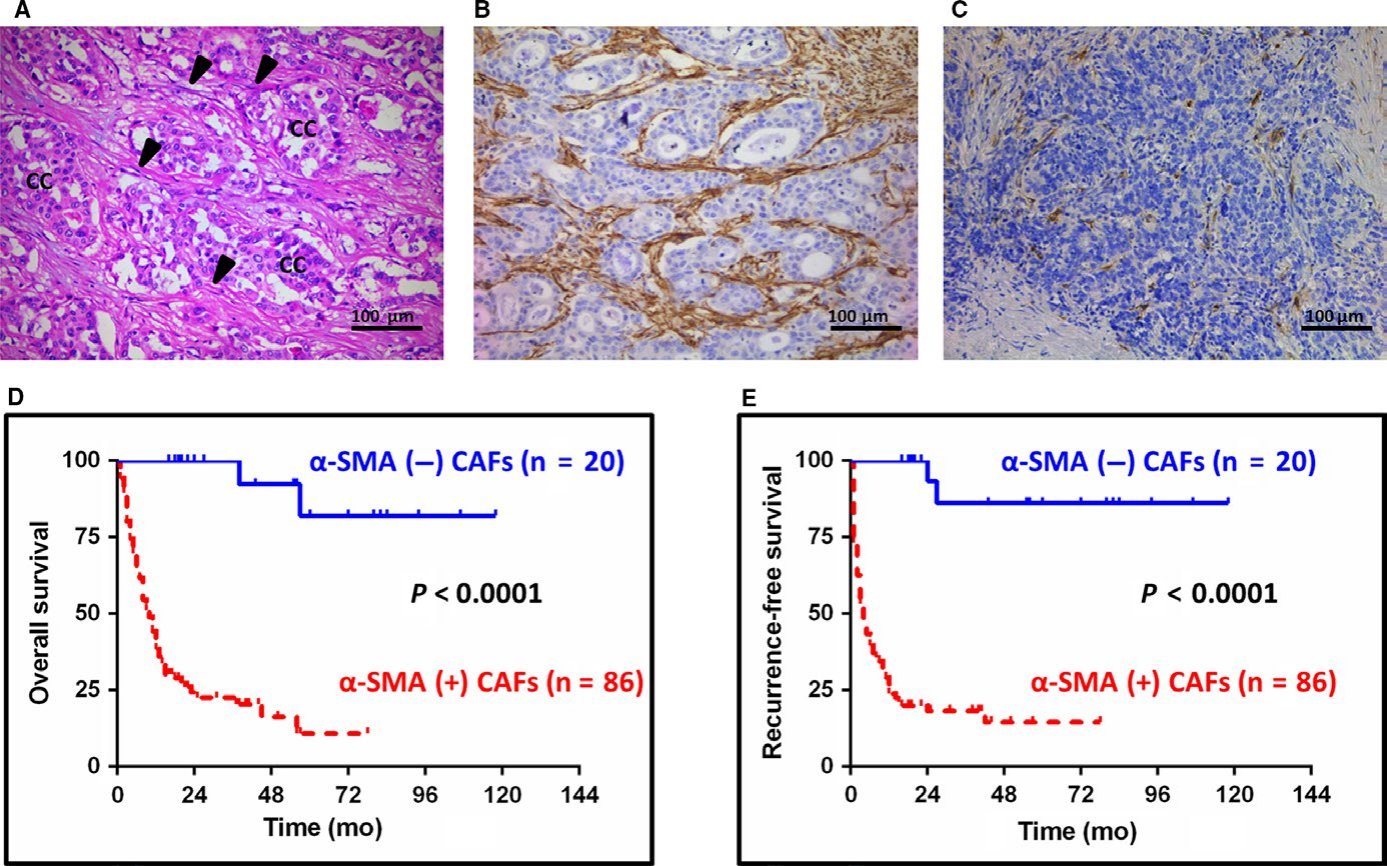
Impact of different expression of α‐SMA in CAFs on survival of patients with ICC. A, Representative H&E staining shows cancer cells (cc) and CAFs (arrowheads) in ICC specimen. B and C, Representative immunohistochemical staining demonstrating high percentage expression of α‐SMA in CAFs of ICC was defined as α‐SMA‐positive CAFs, while low percentage expression of α‐SMA in CAFs was defined as α‐SMA‐negative CAFs (scale bar: 100 μm). D, The overall survival rates of patients with “α‐SMA‐positive CAFs” (n = 86) were much worse than those of patients with “α‐SMA‐negative CAFs” (n = 20; *P *<* *0.0001). E, Patients with “α‐SMA‐positive CAFs” (n = 86) had much worse recurrence‐free survival compared with those of patients with “α‐SMA‐negative CAFs” (n = 20; *P *<* *0.0001)

To further determine the correlation of α‐SMA expression in CAFs with clinicopathological parameters, expression of α‐SMA in CAFs were analyzed (Table 2). Stromal expression of α‐SMA in spindle‐shaped cells was defined as positive when over than 10% of CAFs were stained (Figure 5A‐C). The majority of ICC patients (86/106, 81.1%) enrolled in the study were α‐SMA positively expressed. All patients underwent radical hepatic resection (surgical margin ≥ 2 cm) and nobody was ever infected with fluke by Opisthorchis viverrini. The results revealed that CAFs expressed by α‐SMA were significantly correlated with tumor size (*P *=* *0.024), tumor numbers (*P *=* *0.03), lymph node metastasis (*P *=* *0.012) and histological grade (*P *=* *0.038). However, no association was found in the age, gender, liver background including HBV infection, cirrhosis, and primary sclerosing cholangitis, preoperative level CA19‐9, the presence of vascular invasion and perineural invasion.

**Table 2:** Correlations between α‐SMA expression by CAFs and clinicopathological characteristics in patients with ICC

	α‐SMA expression by CAFs, no. (%)		
Clinicopathological characteristics	Negative	Positive	*P*‐value
Age (years)			0.829
<60	11 (19.6)	45 (80.4)	
≥60	9 (18.0)	41 (82.0)	
Gender			0.174
Female	11 (25)	33 (75)	
Male	9 (14.5)	53 (85.5)	
HBV infection			0.969
Absent	12 (18.8)	52 (81.3)	
Present	8 (19.0)	34 (81.0)	
Primary sclerosing cholangitis			0.381
Absent	19 (20.0)	76 (80.0)	
Present	1 (9.1)	10 (90.9)	
Cirrhosis regardless of etiology			0.675
Absent	16 (19.8)	65 (80.2)	
Present	4 (16.0)	21 (84.0)	
Preoperative CA19‐9 (U/mL)			0.511
<35	5 (15.2)	28 (84.8)	
≥35	15 (20.5)	58 (79.5)	
Tumor size (mm)			0.024
<50	13 (28.9)	32 (71.1)	
≥50	7 (11.5)	54 (88.5)	
Tumor number			0.03
Single	20 (22.5)	69 (77.5)	
Multiple	0 (0)	17 (100)	
Vascular invasion			0.47
Absent	16 (20.5)	62 (79.5)	
Present	4 (14.3)	24 (85.7)	
Lymph node metastasis			0.012
Absent	16 (27.6)	42 (72.4)	
Present	4 (8.3)	44 (91.7)	
Histological grade			0.038
Well or moderate	14 (26.9)	38 (73.1)	
Poor	6 (11.1)	48 (88.9)	
Perineural invasion			0.638
Absent	18 (19.6)	74 (80.4)	
Present	2 (14.3)	12 (85.7)	

### 3.6 The prognostic value of α‐SMA expression by CAFs

We further analyzed clinicopathological variables predicting overall and recurrence‐free survival of ICC. The results of univariate and multivariate analysis for survival were described in Tables 3 and 4. In the univariate analysis, α‐SMA‐positive CAFs, tumor size (≥50 mm) and presence of lymph node metastasis were identified as significant risk factors for overall survival (*P *<* *0.001, *P *=* *0.003 and *P *<* *0.001, respectively). For recurrence‐free survival, the above three parameters combined with tumor number and HBV infection were considered as prognostic factors (*P *<* *0.001, *P *=* *0.004, *P *<* *0.001, *P *=* *0.049 and *P *=* *0.009, respectively). Furthermore, both α‐SMA‐positive CAFs and presence of lymph node metastasis were shown to be statistically significant independent predictors for poor overall (*P *<* *0.001 and *P *=* *0.001, respectively) and recurrence‐free survival (*P *<* *0.001 and *P *=* *0.048, respectively) in multivariate analysis, whereas HBV infection was identified as an independent favorable factor for tumor recurrence (*P *=* *0.001). In addition, we analyzed the influence of α‐SMA‐positive CAFs on survival through Kaplan‐Meier analysis. It was observed that patients with α‐SMA‐positive CAFs had significantly shorter overall survival compared with patients without α‐SMA expression in CAFs (*P *<* *0.0001; Figure 5D). Similarly, patients with α‐SMA‐positive CAFs had a significantly poor prognosis in terms of recurrence‐free survival (*P *<* *0.0001; Figure 5E).

**Table 3:** Univariate Cox proportional hazards analysis in patients with ICC

	Overall survival		Recurrence‐free survival	
Clinicopathological parameter	HR	*P*‐value	HR	*P*‐value
α‐SMA expression by CAFs (Positive vs Negative)	18.826	<0.001	18.386	<0.001
Age (≥60 y vs <60 y)	1.226	0.394	1.302	0.254
Gender (Female vs Male)	1.564	0.074	1.129	0.608
HBV infection (Present vs Absent)	0.674	0.118	0.520	0.009
Primary sclerosing cholangitis (Present vs Absent)	1.131	0.744	1.272	0.501
Cirrhosis regardless of etiology (Present vs Absent)	0.883	0.670	0.888	0.688
Preoperative CA19‐9 (≥35 U/mL vs <35 U/mL)	1.647	0.075	1.499	0.122
Tumor size (≥50 mm vs <50 mm)	2.183	0.003	2.044	0.004
Tumor number (Multiple vs Single)	1.468	0.201	1.752	0.049
Vascular invasion (Present vs Absent)	1.622	0.062	1.286	0.322
Lymph node metastasis (Present vs Absent)	3.161	<0.001	2.816	<0.001
Histological grade (Poor vs Well or Moderate)	1.149	0.561	1.084	0.727
Perineural invasion (Present vs Absent)	1.188	0.615	1.017	0.960

**Table 4:** Multivariate Cox regression model analysis in patients with ICC

	Overall survival			Recurrence‐free survival		
Clinicopathological parameter	HR	95% CI	*P*‐value	HR	95% CI	*P*‐value
α‐SMA expression by CAFs (Positive vs Negative)	15.896	3.711‐68.089	<0.001	20.132	4.755‐85.230	<0.001
Tumor size (≥50 mm vs <50 mm)	1.657	0.984‐2.791	0.058	2.143	1.258‐3.652	0.005
Lymph node Metastasis (Present vs Absent)	2.507	1.492‐4.213	0.001	1.695	1.005‐2.859	0.048
Tumor number (Multiple vs Single)	—	—	—	0.764	0.421‐1.386	0.764
HBV infection (Present vs Absent)	—	—	—	0.391	0.224‐0.681	0.001

## 4 DISCUSSION

Intrahepatic cholangiocarcinoma (ICC) is a highly malignant neoplasm with a dismal prognosis due to insufficient evident symptoms in their early stage. The lack of appropriate medical approaches for ICC calls for further study on its clinical and biological characteristics. Prominent tumor reactive stroma, which interacts between cancer cells and several types of cells and affects cancer invasiveness, is one of the hallmarks of ICC.^8^, ^9^, ^10^, ^11^ CAFs, the main component of tumor stroma, construct tumor‐associated matrix and release a variety of growth factors and chemokines, which modulate apoptosis, migration, and invasion of cancer cells.^21^ In the present study, we isolated human primary CAFs from patients with ICC and discovered that CAFs promoted proliferation, migration, and invasion of cholangiocarcinoma cells in vitro and boosted tumor growth in vivo. Furthermore, it was demonstrated that stroma enriched with α‐SMA‐positive CAFs was associated with poor prognosis of patients with ICC.

Cancer‐associated fibroblasts were isolated by modification of the conventional method^19^ with mixture of trypsin solution and collagenase I from selected samples. In addition, CAFs were purified using anti‐fibroblast microbeads, morphologically identified by the irregular spindle shape and further confirmed by elevated expression level of fibroblast markers through real‐time PCR. Up to present, there is no unique cell marker expressed in all CAFs. CAFs have traditionally been considered as α‐SMA‐positive myofibroblasts,^22^ while other markers have also been used to identify CAFs, including FSP‐1 (fibroblast‐specific protein‐1), FAP (fibroblast activation protein), platelet‐derived growth factor receptor α (PDGFRα), etc.^23^, ^24^, ^25^, ^26^ In this study, the isolated CAFs were immunofluorescence positive for α‐SMA, FSP‐1, FAP, and PDGFR‐β, while NFs were partly stained and low expressed for these markers. Furthermore, the histological findings were similar in human ICC specimens, which further confirmed the characterization of CAFs from ICC.

In order to further study the interactions between CAFs and tumor cells, experiments both in vivo and in vitro were performed. Compared with cancer cells cultured with control medium, CAFs‐conditioned medium significantly promoted the proliferation, migration, and invasion of cholangiocarcinoma cells. Furthermore, CAFs remarkably boosted tumor growth in xenograft model. These findings were in line with several results that connect CAFs and tumor cells in multiple tumors including gastric cancer,^27^ pancreatic cancer,^28^ breast cancer,^29^ etc. However, there has been limited number of studies investigating the effect of CAFs on the molecular functions and biological behavior of cholangiocarcinoma cells. Most recently, Okabe et al^30^ have reported human hepatic stellate or myofibroblastic‐like cell lines (LI90 or LX‐2) significantly increase invasiveness and proliferative activities in cell culture of two human ICC cell lines compared with that observed in control cultures without LI90 or LX‐2 cells. However, neither LI90 nor LX‐2 is CAFs, which could not completely simulate the microenvironment for tumor growth. Our study utilized isolated CAFs to preliminarily identify and confirm the promoting effect of CAFs on cholangiocarcinoma cells both in vitro and in vivo.

Cancer‐associated fibroblasts have been revealed by several studies to facilitate tumor growth and progression through various molecular mechanisms.^31^, ^32^ However, in ICC, only a few studies have explored the signaling pathways involved in the interaction between CAFs and cancer cells. Recently, it has been demonstrated that PDGF‐D secreted by ICC cells promoted recruitment of myofibroblasts through its cognate receptor, PDGFR‐β.^10^ Fingas et al^33^ further emphasized the role of myofibroblast‐derived PDGF‐BB in ICC cell protection through a Hedgehog‐dependent signaling pathway. In addition, HB‐EGF/EGFR pathway^13^ and SDF‐1/CXR4 axis^34^ in the interaction between CAFs and cholangiocarcinoma cells have been proved to promote the progression of human ICC in vitro. Since we have isolated CAFs of ICC and confirmed the promoting effect of CAFs on ICC both in vitro and in vivo preliminarily, further researches investigating the underlying molecular mechanisms between CAFs and cholangiocarcinoma cells will be studied in future.

It has been demonstrated that α‐SMA is widely expressed in CAFs and associated with poor prognosis of patients in various types of cancers, including oral tongue squamous cell carcinoma, hepatocellular carcinoma, non‐small‐cell lung cancer, and colorectal carcinoma.^35^, ^36^, ^37^, ^38^ In our study, it was found that majority of ICC (86/106) are characterized by α‐SMA expression in CAFs. α‐SMA‐positive CAFs in ICC positively correlated with clinicopathological factors including tumor size, tumor number, lymph node metastasis, and histological grade. In addition, patients with α‐SMA‐positive CAFs were characterized by a much worse prognosis compared with α‐SMA‐negative patients. Moreover, α‐SMA‐positive CAFs were found to be an independent prognostic factor. Our results are consistent with studies performed by Chuaysri C^14^ and Okabe H,^30^ which also indicated that α‐SMA expression in fibroblasts may be a predictor of poor prognosis in cholangiocarcinoma. Therefore, it is suggested that α‐SMA‐positive CAFs may be an important factor promoting the progression of ICC.

It has been widely acknowledged that early lymph node metastasis is one of the hallmarks of ICC, which contributes to metastasis of tumor cells and early recurrence.^39^, ^40^ Similarly, our results also showed the presence of lymph node metastasis was an independent risk factor of ICC and positively correlated with α‐SMA expression in CAFs. CAFs have also been discovered exhibiting several cellular phenotypic switches including epithelial‐to‐mesenchymal (EMT),^41^ mesenchymal‐to‐epithelial (MET),^42^ and endothelial‐to‐mesenchymal (EndMT) transitions.^43^, ^44^ This renders us to emphasize that CAFs can adopt different cell fate to complete the transition, except for mesenchymal‐to‐endothelial (MEndT) transition, which is yet to be discovered in tumor entities. In 2014, Ubil et al^45^ demonstrated that cardiac fibroblasts could transform into vascular endothelial cells after ischemic cardiac injury, thereby participating in neovascularization to promote cardiac repair, so‐called the initial discovery of MEndoT. Another study^46^ confirmed a new origin of Kaposi sarcomas transformed from MSCs through a novel pathway of mesenchymal‐to‐endothelial transition (MEndT). Based on the phenotypic transformations of CAFs, there might be a potential role of CAFs in the correlation with lymphatic endothelial cells and lymphangiogenesis in tumor entities. However, up to date, there is no conclusive evidence of CAFs to lymphatic connection in ICC, which awaits our future researches.

In this study, we have successfully isolated and purified the primary human CAFs in vitro from resected ICC samples. The isolated cells expressed fibroblast markers and are exclusively observed in cancerous stroma. Furthermore, the isolated CAFs were found to promote the progression of cholangiocarcinoma cells both in vitro and in vivo. In addition, α‐SMA‐positive expression of CAFs predicted unfavorable prognosis in patients with ICC and showed correlation with presence of lymph node metastasis. Further researches regarding the potential mechanism between CAFs and cholangiocarcinoma cells as well as contribution of CAFs in lymph node metastasis need to be performed.

## CONFLICT OF INTERESTS

The authors declare no conflict of interest.
